# A retrospective study of amrubicin monotherapy for the treatment of relapsed small cell lung cancer in elderly patients

**DOI:** 10.1007/s00280-017-3403-9

**Published:** 2017-07-31

**Authors:** Hisao Imai, Tomohide Sugiyama, Tomohiro Tamura, Hiroyuki Minemura, Kyoichi Kaira, Kenya Kanazawa, Hiroshi Yokouchi, Takashi Kasai, Takayuki Kaburagi, Koichi Minato, Gunma-Ibaraki-Fukushima-Tochigi (GIFT) group

**Affiliations:** 1 Division of Respiratory Medicine, Gunma Prefectural Cancer Center, 617-1, Takahayashinishi, Ohta, Gunma 373-8550 Japan; 2 Division of Thoracic Oncology, Tochigi Cancer Center, Utsunomiya, Tochigi Japan; 3 Division of Respiratory Medicine, Ibaraki Prefectural Central Hospital, Kasama, Ibaraki Japan; 4 Department of Pulmonary Medicine, Fukushima Medical University, Fukushima, Japan; 5 Department of Oncology Clinical Development, Gunma University Graduate School of Medicine, Maebashi, Gunma Japan; 6 Clinical Oncology Center, Fukushima Medical University Hospital, Fukushima, Japan

**Keywords:** Small cell lung cancer, Amrubicin, Relapse, Sensitive, Refractory, Elderly patients

## Abstract

**Purpose:**

Amrubicin is one of the most active chemotherapeutic drugs for small cell lung cancer (SCLC). Previous studies reported its effectiveness and severe hematological toxicity. However, the efficacy of amrubicin monotherapy in elderly patients with SCLC has not been described. The objective of this study was to investigate the feasibility of amrubicin monotherapy in elderly patients and its efficacy for relapsed SCLC.

**Methods:**

A retrospective cohort study design was used. We retrospectively evaluated the clinical effects and adverse events of amrubicin treatment in elderly (≥70 years) SCLC patients with relapsed SCLC.

**Results:**

Between November 2003 and September 2015, 86 patients (aged ≥70 years) received amrubicin monotherapy for relapsed SCLC at four institutions. There were 42 cases of sensitive relapse (S) and 44 of refractory relapse (R). S cases with median age of 75 years (range 70–85 years) and R cases with median age of 74 years (range 70–84 years) were included in our analysis. The median number of treatment cycles was three (range 1–9), and the response rate was 33.7% (40.5% in the S and 27.2% in the R cases). Median progression-free survival time was 4.0 months in the S and 2.7 months in the R patients (*p* = 0.013). Median survival time from the start of amrubicin therapy was 7.6 months in the S and 5.5 months in the R cases (*p* = 0.26). The frequencies of grade ≥3 hematological toxicities were as follows: leukopenia, 60.4%; neutropenia, 74.4%; anemia, 11.6%; thrombocytopenia, 16.2%; and febrile neutropenia, 17.4%. Treatment-related death was observed in one patient.

**Conclusion:**

Although hematological toxicities, particularly neutropenia, were severe, amrubicin showed favorable efficacy, not only in the S but also in the R cases, as shown in previous studies. Amrubicin could be a preferable standard treatment in elderly patients with relapsed SCLC. These results warrant further evaluation of amrubicin in elderly patients with relapsed SCLC by a prospective trial.

**Electronic supplementary material:**

The online version of this article (doi:10.1007/s00280-017-3403-9) contains supplementary material, which is available to authorized users.

## Introduction

Small cell lung cancer (SCLC) accounts for approximately 15% of all lung cancer cases and is characterized by an aggressive nature and rapid growth [1]. Most patients with SCLC will, despite administration of first-line chemotherapy, experience recurrence of their disease, ultimately leading to death due to complications caused by extensive systemic metastases. The poor outcomes faced by patients with relapsed SCLC highlight the urgent need for research and development of more effective chemotherapeutic agents.

Advanced age is associated with an increased risk of lung cancer. The increase in global life expectancy has resulted in a corresponding increase in the incidence of lung cancer. The elderly population has been disproportionately affected, with considerable increases in the disease incidence in this age group. More than half of lung cancer cases are diagnosed in patients older than 65 years, which is the lower limit for defining “elderly” in epidemiological studies [2]. Approximately, 30–40% of patients with SCLC are ≥70 years old at diagnosis [3], and the understanding of how SCLC treatment should be tailored for elderly patients is becoming increasingly important. The Japan Clinical Oncology Group (JCOG) recommended that carboplatin plus etoposide was an active and less toxic regimen in elderly patients with SCLC [4]. There are, at present, no recommended chemotherapy agents or regimens for relapsed SCLC in elderly patients. Further complicating the treatment of these patients is their tendency to poorly tolerate chemotherapy and the requirement of clinical management based on individual parameters such as performance status, extent of metastatic disease, quality of life and laboratory data [5–7]. Whether standard chemotherapy in elderly patients is always safe for use in clinical practice is unclear [8].

Previously treated SCLC patients can be divided into two groups: (a) refractory cases, where first-line chemotherapy failed, or less than 90 days passed between the end of chemotherapy and recurrence of the disease; and (b) sensitive cases, where there was a response to first-line chemotherapy and relapse after a treatment-free interval of at least 90 days [9, 10]. Sensitive cases are more likely to respond to second-line chemotherapy than refractory cases. Regardless, second-line chemotherapy against SCLC is disappointing [11, 12]. Refractory cases present a distinct clinical challenge, as they rarely respond to second-line, single-agent chemotherapy and may only respond to non-cross-resistant combination chemotherapy [13]. Despite the chemoresistance demonstrated by refractory SCLC, Murakami et al. reported that the anti-tumor activity of amrubicin was promising, and amrubicin could be considered an effective and safe treatment option for refractory SCLC in non-elderly patients [14].

Amrubicin and its active metabolite amrubicinol are inhibitors of DNA topoisomerase II, which exert cytotoxic effects by stabilizing a topoisomerase II-mediated cleavable complex, and not by DNA intercalation [15]. Amrubicinol was 5–100 times more active than amrubicin [16]. In a phase II study of amrubicin using a schedule of 45 mg/m^2^ on days 1–3 every 3 weeks in 33 previously untreated patients with extensive-stage SCLC, an overall response rate of 76% and a complete response (CR) rate of 9% was reported. Moreover, median survival time (MST) was 11.7 months in that single-agent phase II study of amrubicin [17]. In a phase II study evaluating the activity of amrubicin in relapsed SCLC, the response rate and MST were 52% and 11.6 months, and 50% and 10.3 months in sensitive and refractory relapse, respectively [18]. The most frequent drug-related adverse event is myelosuppression, and the incidence of non-hematological toxicities is low [18–23].

The toxicity and effect of amrubicin monotherapy in elderly patients who have been previously treated for lung cancer have not been fully evaluated. We therefore retrospectively assessed the safety and activity of amrubicin among elderly patients who had previously been treated for small cell lung cancer.

## Patients and methods

In this retrospective study, patient records were included if the patient was ≥70 years, diagnosed with relapsed SCLC and treated with amrubicin monotherapy between November 2003 and September 2015 at one of four Japanese institutions (Gunma Prefectural Cancer Center, Tochigi Cancer Center, Ibaraki Prefectural Central Hospital, and Fukushima Medical University). Histological diagnosis and staging of SCLC were based on the World Health Organization classification and the tumor–node–metastasis staging system [24], respectively. Eligibility criteria were histologically or cytologically confirmed SCLC, unresectable stage III/IV disease at first-line treatment and treatment in the first-line setting with a platinum-based combination chemotherapy. Before receiving therapy, each patient underwent physical examination, chest radiography, thorax and abdomen computed tomography, bone scintigraphy or ^18^F-fluorodeoxyglucose positron emission tomography, and brain computed tomography or magnetic resonance imaging to determine the TNM stage. In this study, patients who responded to initial chemotherapy and relapsed >3 months after chemotherapy were defined as sensitive relapse (S) patients, while patients who did not respond to initial chemotherapy or relapsed within 3 months were defined as refractory relapse (R) patients. For the identified and selected subjects, a clinical chart search was performed at each hospital. Institutional review boards of each institution approved the study protocol, and the requirement for written informed consent was waived, given this study’s retrospective nature. All patients were amrubicin monotherapy naïve, and amrubicin was dissolved in 20 ml of normal saline and administered intravenously as a 5-min infusion at a dose of 30–45 mg/m^2^ on days 1–3 every 3 or 4 weeks. Granulocyte colony-stimulating factor (G-CSF) was administered as a therapeutic intervention at the physician’s discretion, but it was not mandatory as a prophylactic agent against leukopenia or neutropenia. Treatment continued until disease progression, the appearance of intolerable toxicity, or withdrawal of consent.

The best overall response and maximum tumor shrinkage were recorded as the tumor responses. Radiographic tumor responses were defined according to the Response Evaluation Criteria in Solid Tumors, version 1.1 [25]: complete response (CR), the disappearance of all target lesions; partial response (PR), a decrease in the sum of the target lesion diameters by at least 30% compared to baseline; progressive disease (PD), an increase of at least 20% in the sum of the target lesion diameters compared to the smallest sum during the study; and stable disease (SD), insufficient shrinkage or expansion to qualify as PR or PD. PFS was calculated from the start of treatment until PD or death from any cause, and OS was recorded from the first day of treatment until death, or was censored on the date of the last follow-up. The survival curves were calculated using the Kaplan–Meier method. Adverse events that were associated with amrubicin monotherapy were graded according to the Common Terminology Criteria for Adverse Events (version 4.0). After failure of amrubicin monotherapy, patients were permitted any subsequent treatment(s) desired by themselves, including continuation of amrubicin treatment. All statistical analyses were performed using JMP, version 11.0, for Windows (SAS Institute, Cary, NC, USA).

## Results

### Patients characteristics

From November 2003 and September 2015 at four institutions, a total of 86 patients were treated with a single-agent regimen of amrubicin. Patient baseline characteristics are shown in Table 1. Forty-two patients in the sensitive group and 44 in the refractory group were assessable for response, survival and safety. Men accounted for the bulk of the patients (76, 88.3%), and the median age of the entire group was 75 years (range 70–85 years). The bulk of the patient group (71, 82.6%) had only received one previous line of chemotherapy prior to the use of amrubicin, and the remaining patients had received two prior lines of treatment. All patients had been pretreated using chemotherapy containing any type of topoisomerase inhibitor: topoisomerase I inhibitor (irinotecan or topotecan), *n* = 5; topoisomerase II inhibitor (etoposide), *n* = 71; and both topoisomerase I and II inhibitors, *n* = 8.

**Table 1:** Baseline patient characteristics (*n* = 86)

	Sensitive group (*n* = 42)	Refractory group (*n* = 44)
Sex		
Male (%)	36 (85.7)	40 (90.9)
Female (%)	6 (14.3)	4 (9.0)
Age (years)		
70–74 (%)	16 (38.1)	24 (54.5)
75–79 (%)	17 (40.5)	9 (20.5)
≥80 (%)	9 (21.4)	11 (25.0)
Performance status		
0–1	35 (83.3)	34 (77.3)
2	7 (16.7)	9 (20.5)
≥3	0 (0.0)	1 (2.3)
Disease extent		
Limited disease (%)	19 (45.2)	11 (25.0)
Extensive disease (%)	23 (54.7)	33 (27.0)
Prior therapy		
Chemotherapy alone (%)	30 (71.4)	41 (93.9)
Chemotherapy and thoracic radiation (%)	12 (28.6)	2 (4.5)
Chemotherapy and surgery (%)	0 (0.0)	1 (2.3)

### Response and treatment delivery

Table 2 shows the results of the response and treatment delivery. There were no complete response (CR), 17 partial response (PR), 9 stable disease (SD) and 14 progressive disease (PD) cases in the S group (response rate 40.5%) and no CR, 12 PR, 12 SD and 20 PD cases in the R group (response rate 27.2%). The median number of treatment cycles was three (range 1–9) in the S group and two (range 1–6) in the R group. None of the patients received amrubicin at a dose of 45 mg/m^2^ per day, while 16 patients received 40 mg/m^2^ per day, 23 patients received 35 mg/m^2^ per day and 3 patients received 30 mg/m^2^ per day in the S group. Two patients received amrubicin at a dose of 45 mg/m^2^ per day, 13 patients received 40 mg/m^2^ per day, 28 patients received 35 mg/m^2^ per day and 1 patient received 30 mg/m^2^ per day in the R group. Dose reduction was more frequent at a dose of 40 mg/m^2^ per day or more than those at 35 mg/m^2^ per day or less [occurring in 47.6% (10/21) vs. 17.0% (8/47) of patients].

**Table 2:** Tumor response to therapy and treatment delivery

	Sensitive group (*n* = 42)	Refractory group (*n* = 44)	Total
Response			
CR	0	0	0
PR	17	12	29
SD	9	12	21
PD	14	20	34
NE	2	0	2
Response rate (%)	40.5	27.2	33.7
Disease control rate (%)	61.9	54.5	58.1
No. of treatment cycles			
Median	3	2	3
Range	1–9	1–6	1–9
Starting dose (mg/m^2^ per day)			
45	0	2	2
40	16	13	29
35	23	28	51
30	3	1	4
Dose reduction			
Starting dose 30–35 mg/m^2^ per day			
Yes/no	5/21	3/26	8/47
Starting dose 40–45 mg/m^2^ per day			
Yes/no	5/11	5/10	10/21

*CR* complete response, *PR* partial response, *SD* stable disease, *PD* progressive disease, *NE* not evaluate

### Survival

**Fig. 1:**
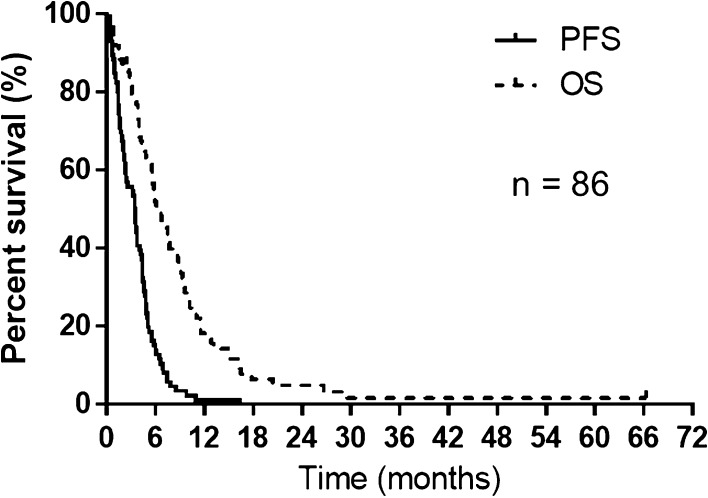
Kaplan–Meier estimates for progression-free and overall survival of the entire study population (*n* = 86). Median progression-free and overall survival was 3.4 and 6.1 months, respectively

**Fig. 2:**
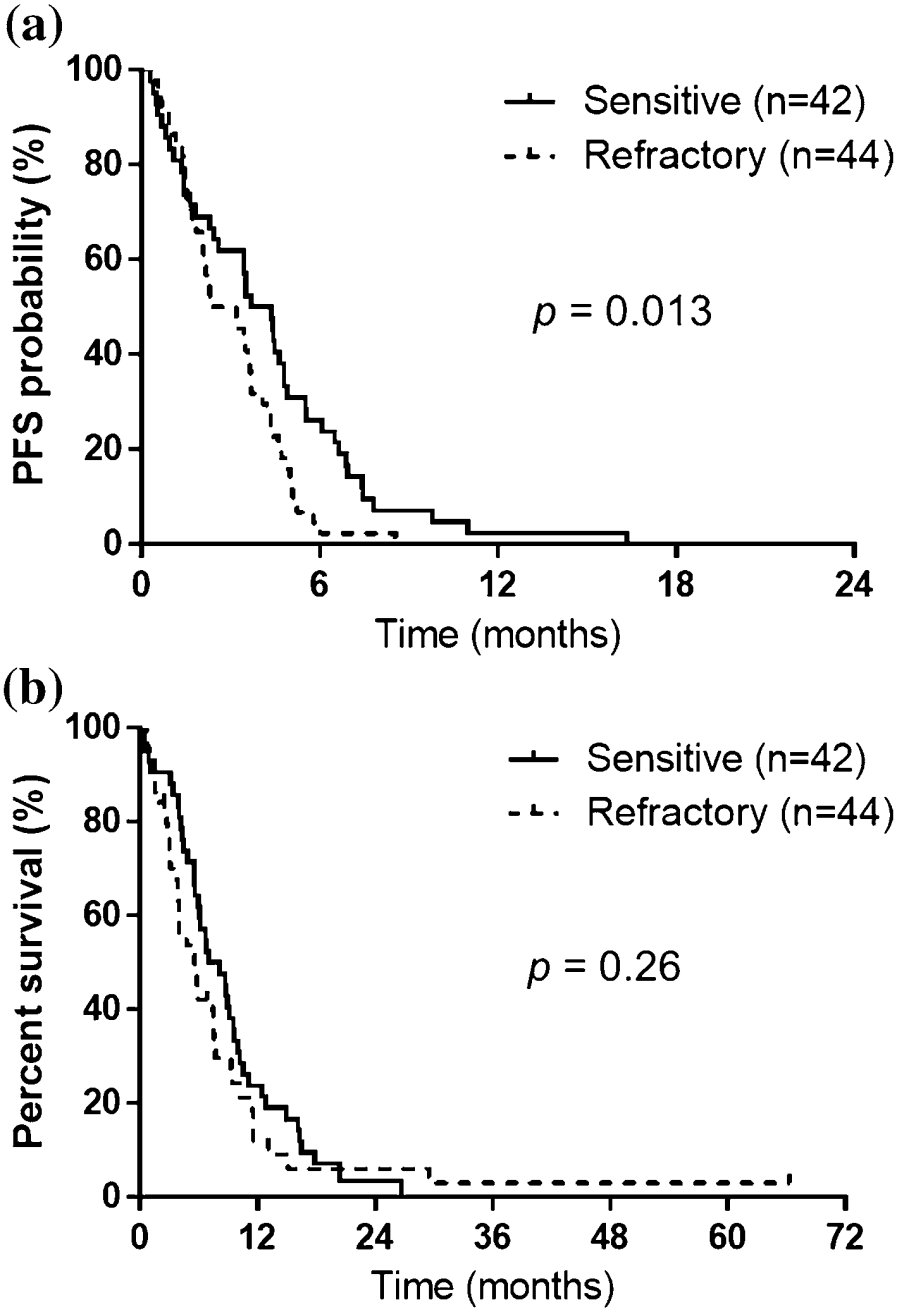
**a** Median progression-free survival (PFS) in the sensitive- and refractory-relapsed patients was 4.0 and 2.7 months, respectively (*p* = 0.013). **b** Median overall survival (OS) of sensitive- and refractory-relapsed patients was 7.6 and 5.5 months, respectively (*p* = 0.26)

Median progression-free survival (PFS) was 3.4 months for all patients (Fig. 1). Median overall survival (OS) from the first amrubicin administration for all patients was 6.1 months (Fig. 1). PFS was significantly better in the S group (Fig. 2a). Median PFS was 4.0 months in the S group and 2.7 months in the R group, respectively (*p* = 0.013). OS according to relapse pattern is shown in Fig. 2b. Median OS was 7.6 months in the S group and 5.5 months in the R group, respectively (*p* = 0.26). Univariate and multivariate analyses showed that the relapse pattern (sensitive or refractory) was an independent prognostic factor for PFS. Univariate and multivariate analyses showed that: a PS of 0–1 or 2–4 for amrubicin was an independent prognostic factor for OS (Table 3); the relapse pattern (sensitive or refractory) was an independent prognostic factor for PFS in patients with a good PS (PS 0–1; Supplement Table 1); a PS of 0–1 or 2–4 for amrubicin was an independent prognostic factor for OS in sensitive relapse patients (Supplement Table 2). Only the univariate analysis showed that an age of 70–74 or ≥75 years for amrubicin was a prognostic factor for OS in refractory relapse patients (Supplement Table 3).

**Table 3:** Univariate and multivariate analyses for progression-free survival and overall survival

Factors	Median PFS (months)	Univariate analysis			Multivariate analysis			Median OS (months)	Univariate analysis			Multivariate analysis		
		PFS			PFS				OS			OS		
		HR	95% CI	*p* value	HR	95% CI	*p* value		HR	95% CI	*p* value	HR	95% CI	*p* value
Sex														
Male/female	3.4/2.9	0.95	0.51–1.96	0.88	0.92	0.48–1.95	0.82	6.1/8.1	1.26	0.64–2.85	0.51	1.31	0.64–3.04	0.47
Age (years)														
70–74/≥75	3.5/3.4	1.16	0.75–1.79	0.47	1.07	0.67–1.70	0.74	6.1/6.4	0.82	0.52–1.28	0.38	0.79	0.48–1.29	0.36
PS														
0–1/2–4	3.5/2.3	0.84	0.51–1.49	0.55	0.89	0.53–1.59	0.69	7.4/4.5	0.43	0.24–0.80	**0.009**	0.47	0.26–0.89	**0.022**
Dose (mg/m^2^ per day)														
30–35/40–45	3.4/3.6	1.11	0.72–1.75	0.63	1.11	0.69–1.80	0.65	5.5/7.4	1.38	0.87–2.22	0.16	1.31	0.80–2.17	0.27
Relapse pattern														
Sensitive/refractory	4.0/2.7	0.57	0.36–0.89	**0.014**	0.58	0.36–0.92	**0.022**	7.6/5.5	0.77	0.49–1.21	0.27	0.73	0.45–1.20	0.22

Bold-type *p* values are statistically significant (*p* < 0.05)

*PFS* progression-free survival, *OS* overall survival, *HR* hazard ratio, *95% CI* 95% confidence interval, *PS* performance status

### Toxicity

Drug-related adverse events for all patients are shown in Table 4. All 86 patients were evaluated for toxicity. The most frequent drug-related adverse event was myelosuppression. Grade 3 or 4 neutropenia was seen in 74.4% of patients, and grade 3 or 4 leukopenia occurred in 60.4%. Febrile neutropenia was observed in 15 patients (17.4%). Grade 3 or 4 anemia occurred in 10 patients (11.6%) and grade 3 or 4 thrombocytopenia was reported in 14 patients (16.2%). The incidence of non-hematologic toxicities was low. The most frequent grade 3 or 4 non-hematologic toxicities included anorexia (5.8%) and infection (5.8%). Grade 1 pneumonitis was seen in one patient. No cardiotoxicity was noted. Treatment-related death occurred in one patient. The patient suffered from bacterial lung infection.

**Table 4:** Patients with drug-related adverse events (CTCAE v4.0)

Event	Gr.1	Gr. 2	Gr.3	Gr.4	Gr.5	≥Gr.3	%
Leukopenia							
Total	5	19	33	19	–	52	60.4
Dose of 30–35 mg/m^2^/day	4	13	21	10	–	31	56.3
Dose of 40–45 mg/m^2^/day	1	6	12	9	–	21	67.7
Neutropenia							
Total	4	8	26	38	–	64	74.4
Dose of 30–35 mg/m^2^/day	4	6	14	24	–	38	60.1
Dose of 40–45 mg/m^2^/day	0	2	12	14	–	26	83.8
Anemia							
Total	18	21	7	3	0	10	11.6
Dose of 30–35 mg/m^2^/day	14	14	4	1	0	5	9.1
Dose of 40–45 mg/m^2^/day	4	7	3	2	0	5	16.1
Thrombocytopenia							
Total	13	19	8	6	–	14	16.2
Dose of 30–35 mg/m^2^/day	11	7	4	3	–	7	12.7
Dose of 40–45 mg/m^2^/day	2	12	4	3	–	7	22.5
Febrile neutropenia							
Total	–	–	14	1	0	15	17.4
Dose of 30–35 mg/m^2^/day	–	–	8	0	0	8	14.5
Dose of 40–45 mg/m^2^/day	–	–	6	1	0	7	33.3
Nausea/vomiting	17	10	1	0	0	1	1.1
Anorexia	10	11	5	0	0	5	5.8
Malaise	11	7	–	–	–	0	0
Infection	0	1	4	0	1	5	5.8
Pneumonitis	1	0	0	0	0	0	0

*Gr* grade

Analyzing myelosuppression according to dose of administration, hematologic toxicities occurring at a dose of 35 mg/m^2^ per day or less were milder than those at 40 mg/m^2^ per day or more (Table 4). The frequencies of grade 3 or 4 hematologic toxicities in patients receiving 35 mg/m^2^ per day or less versus those receiving 40 mg/m^2^ per day or more were as follows: neutropenia, 60.1 versus 83.8%; leukopenia, 56.3 versus 67.7%; anemia, 9.1 versus 16.1%; and thrombocytopenia, 12.7 versus 22.5%. Febrile neutropenia occurred in 14.5% of patients when the dose was 35 mg/m^2^ per day or less and in 33.3% of patients when the dose was 40 mg/m^2^ per day or more.

## Discussion

This retrospective study assessed the effect and safety of amrubicin monotherapy for the treatment of recurrent or refractory SCLC, in patients who had previously been treated with a platinum-based regimen. Amrubicin demonstrated no new safety signals in this patient population and was acceptable for elderly patients with previously treated SCLC.

The number of elderly patients with SCLC will increase with the swiftly growing geriatric population [26, 27]. Elderly patients with good PS and normal organ functions tend to be treated with regimens analogous with those of younger patients, but some studies suggest that elderly patients even with good PS and normal organ function may be at greater risk for severe toxicity than younger counterparts [28, 29]. Therefore, non-cisplatin regimens, such as carboplatin and etoposide, have become one of the standard chemotherapy regimens for elderly patients with SCLC [30]. Treatment options for elderly patients with recurrent or refractory SCLC remain limited. Amrubicin is often delivered to patients with refractory or recurrent SCLC, but whether amrubicin monotherapy for elderly patients with previously treated SCLC is clinically safe has not been evaluated. This is the first study of the safety and benefit of amrubicin for elderly patients with previously treated SCLC.

Following front-line chemotherapy for SCLC, chemoresistance is common, and few subsequent treatment options exist [31]. Our analysis shows that amrubicin monotherapy is active against relapsed SCLC. The activity of second-line treatments usually depends on tumor responsiveness to first-line treatment: namely, whether the tumor is sensitive or refractory. Amrubicin and topotecan are agents that have demonstrated efficacy in the second-line setting [21, 32]. In a Japanese randomized phase II study comparing amrubicin with topotecan, the response rate in the amrubicin arm was better in the S group (53%) compared to the R group (17%) [21]. In the study, the median PFS and OS in the amrubicin arm were 3.9 and 9.9 months in the S group, and 2.6 and 5.3 months in the R cases, respectively. Furthermore, in a randomized phase III study comparing amrubicin with topotecan, the response rate, the median PFS and the median OS in the amrubicin arm were better with 40.9%, 5.5 and 9.2 months in the S and 20.1%, 2.8 and 6.2 months in the R cases, respectively [32]. Considering that R is extremely chemoresistant, the anti-tumor activity of amrubicin is considerably promising. Onoda et al. also described a single-arm phase II study of amrubicin 40 mg/m^2^ for relapsed SCLC [18]. The ORR was 52% (sensitive relapse, 52%; refractory relapse, 50%). The median PFS and MST in the sensitive relapse and the refractory relapse were 4.2 and 2.6 months, and 11.6 and 10.3 months, respectively. In the above trials of single-agent activity, response rates, median PFS and median OS for refractory disease were unsatisfactory. Although inferior to S, a response rate of 27.2% was observed in the R group in our study. Although the present study was retrospective, the response rate in the R group was reasonable. OS was not significantly different between S and R patients. However, PFS was significantly more favorable in the S patients (4.0 vs. 2.7 months, *p* = 0.013). The response rate and median PFS of SCLC in our study seemed to be equal to that of these previous reports. Although the response rates and median PFS were similar to previous reports, the results of OS were slightly lower than those reported in the studies [18, 21, 32]. These differences may be due to differences of patient characteristics or other biases. Previous relapsed SCLC studies of amrubicin monotherapy included only a small percentage of poor PS and elderly patients and a large percentage of treatment as second-line setting. Furthermore, median OS was similar in the two groups (7.6 months in the sensitive group and 5.5 months in the refractory group, *p* = 0.26; Fig. 2b). These results suggest that amrubicin may be a useful new addition to treatment strategies for chemotherapy-resistant/refractory elderly patients. Likewise, our results of elderly patients are not inferior to those of previous reports of non-elderly patients.

Multivariate analysis demonstrated that PS of 0–1 or 2–4 for amrubicin was an independent prognostic factor for OS. The result was consistent with a previous study that included non-elderly patients [33]. These findings suggest that amrubicin monotherapy might be beneficial for elderly relapsed SCLC patients of good PS in OS. Furthermore, multivariate analysis demonstrated that the relapse pattern (sensitive or refractory) was an independent prognostic factor for PFS in all patients and good PS patients. The log-rank tests confirmed that PFS was significantly associated with the relapse pattern (sensitive or refractory) in all patients (Fig. 2a).

The toxicity profile of amrubicin noted in our analysis was acceptable, in accordance with previous phase II and III trials that noted myelosuppression as the major toxic effect [18, 21, 32]. Almost all hematologic adverse effects were manageable. Non-hematologic toxicity was generally mild. The frequency of grade ≥3 hematologic toxicities occurring at a dose of 40 mg/m^2^ per day or more was much more than that at 35 mg/m^2^ per day or less. Furthermore, dose reduction was more frequent at a dose of 40 mg/m^2^ per day or more than that at 35 mg/m^2^ per day or less (Table 2). Univariate and multivariate analyses showed that the initial dose (30–35 or 40–45 mg/m^2^ per day) was not independent prognostic factors for PFS and OS (Table 3). A previous study indicated that the patients treated with amrubicin 35 mg/m^2^ seemed to achieve similar efficacy with less toxicity than those with amrubicin 40 mg/m^2^ in relapsed SCLC [23]. Besides, Okamoto et al. conducted a phase I and pharmacokinetic study of amrubicin in previously treated patients with SCLC and non-small cell lung cancer (NSCLC) and described a recommended dose of amrubicin at 35 mg/m^2^/day for three consecutive days every 3 weeks [34]. Thus, the patients treated with amrubicin 35 mg/m^2^ might achieve similar efficacy with less toxicity than those with a dose of 40 mg/m^2^ per day or more in relapsed SCLC. A dose of 35 mg/m^2^ might be reasonable for elderly relapsed SCLC. A single patient developed pneumonitis, but this was reversible with steroid therapy. However, treatment-related death was observed in one patient due to infection. Experimentally and clinically, long-term treatment with doxorubicin is well known to cause cardiomyopathy. In contrast, repeated administration of amrubicin in the present series did not cause any cardiotoxicity. These findings suggest that the toxicity profile of amrubicin may be acceptable in the treatment of relapsed SCLC.

In conclusion, this retrospective study provides evidence that amrubicin monotherapy may be an effective and tolerable regimen for elderly patients with previously treated SCLC. The information presented herein might provide a new direction for clinical research on the treatment of elderly patients with SCLC after one or more chemotherapy regimens.

## Electronic supplementary material

Below is the link to the electronic supplementary material.

Supplementary material 1 (DOCX 24 kb)

## References

[1] Govindan R, Page N, Morgensztern D (2006). Changing epidemiology of small-cell lung cancer in the United States over the last 30 years: analysis of the surveillance, epidemiologic, and end results database. J Clin Oncol.

[2] Davidoff AJ, Tang M, Seal B, Edelman MJ (2010). Chemotherapy and survival benefit in elderly patients with advanced non-small-cell lung cancer. J Clin Oncol.

[3] Pallis AG, Shepherd FA, Lacombe D, Gridelli C (2010). Treatment of small-cell lung cancer in elderly patients. Cancer.

[4] Okamoto H, Watanabe K, Nishiwaki Y (1999). Phase II study of area under the plasma-concentration-versus-time curve-based carboplatin plus standard-dose intravenous etoposide in elderly patients with small-cell lung cancer. J Clin Oncol.

[5] Sekine I, Fukuda H, Kunitoh H, Saijo N (1998). Cancer chemotherapy in the elderly. Jpn J Clin Oncol.

[6] Gridelli C, Shepherd FA (2005). Chemotherapy for elderly patients with non-small cell lung cancer: a review of the evidence. Chest.

[7] Chrischilles EA, Pendergast JF, Kahn KL (2010). Adverse events among the elderly receiving chemotherapy for advanced non-small-cell lung cancer. J Clin Oncol.

[8] Li J, Chen P, Dai CH, Li XQ, Bao QL (2009). Outcome and treatment in elderly patients with small cell lung cancer: a retrospective study. Geriatr Gerontol Int.

[9] Ebi N, Kubota K, Nishiwaki Y (1997). Second-line chemotherapy for relapsed small cell lung cancer. Jpn J Clin Oncol.

[10] Giaccone G, Donadio M, Bonardi G, Testore F, Calciati A (1988). Teniposide in the treatment of small-cell lung cancer: the influence of prior chemotherapy. J Clin Oncol.

[11] Jackman DM, Johnson BE (2005). Small-cell lung cancer. Lancet.

[12] Kim YH, Mishima M (2011). Second-line chemotherapy for small-cell lung cancer (SCLC). Cancer Treat Rev.

[13] Postmus PE, Smit EF, Kirkpatrick A, Splinter TA (1993). Testing the possible non-cross resistance of two equipotent combination chemotherapy regimens against small-cell lung cancer: a phase II study of the EORTC Lung Cancer Cooperative Group. Eur J Cancer.

[14] Murakami H, Yamamoto N, Shibata T (2014). A single-arm confirmatory study of amrubicin therapy in patients with refractory small-cell lung cancer: Japan Clinical Oncology Group Study (JCOG0901). Lung Cancer.

[15] Tani N, Yabuki M, Komuro S, Kanamaru H (2005). Characterization of the enzymes involved in the in vitro metabolism of amrubicin hydrochloride. Xenobiotica.

[16] Yamaoka T, Hanada M, Ichii S, Morisada S, Noguchi T, Yanagi Y (1998). Cytotoxicity of amrubicin, a novel 9-aminoanthracycline, and its active metabolite amrubicinol on human tumor cells. Jpn J Cancer Res.

[17] Yana T, Negoro S, Takada M (2007). Phase II study of amrubicin in previously untreated patients with extensive-disease small cell lung cancer: West Japan Thoracic Oncology Group (WJTOG) study. Invest New Drugs.

[18] Onoda S, Masuda N, Seto T (2006). Phase II trial of amrubicin for treatment of refractory or relapsed small-cell lung cancer: thoracic oncology research group study 0301. J Clin Oncol.

[19] Kurata T, Okamoto I, Tamura K, Fukuoka M (2007). Amrubicin for non-small-cell lung cancer and small-cell lung cancer. Invest New Drugs.

[20] Igawa S, Ryuge S, Fukui T (2010). Amrubicin for treating elderly and poor-risk patients with small-cell lung cancer. Int J Clin Oncol.

[21] Inoue A, Sugawara S, Yamazaki K (2008). Randomized phase II trial comparing amrubicin with topotecan in patients with previously treated small-cell lung cancer: North Japan Lung Cancer Study Group Trial 0402. J Clin Oncol.

[22] Shimokawa T, Shibuya M, Kitamura K (2009). Retrospective analysis of efficacy and safety of amrubicin in refractory and relapsed small-cell lung cancer. Int J Clin Oncol.

[23] Kaira K, Sunaga N, Tomizawa Y (2010). A phase II study of amrubicin, a synthetic 9-aminoanthracycline, in patients with previously treated lung cancer. Lung Cancer.

[24] Goldstraw P, Crowley J, Chansky K (2007). The IASLC lung cancer staging project: proposals for the revision of the TNM stage groupings in the forthcoming (seventh) edition of the TNM classification of malignant tumours. J Thorac Oncol.

[25] Eisenhauer EA, Therasse P, Bogaerts J (2009). New response evaluation criteria in solid tumours: revised RECIST guideline (version 1.1). Eur J Cancer.

[26] Stephens RJ, Johnson DH (2000). Treatment and outcomes for elderly patients with small cell lung cancer. Drugs Aging.

[27] Sekine I, Yamamoto N, Kunitoh H (2004). Treatment of small cell lung cancer in the elderly based on a critical literature review of clinical trials. Cancer Treat Rev.

[28] Oshita F, Kurata T, Kasai T (1995). Prospective evaluation of the feasibility of cisplatin-based chemotherapy for elderly lung cancer patients with normal organ functions. Jpn J Cancer Res.

[29] Jara C, Gomez-Aldaravi JL, Tirado R, Meseguer VA, Alonso C, Fernandez A (1999). Small-cell lung cancer in the elderly—is age of patient a relevant factor?. Acta Oncol.

[30] Okamoto H, Watanabe K, Kunikane H (2007). Randomised phase III trial of carboplatin plus etoposide vs split doses of cisplatin plus etoposide in elderly or poor-risk patients with extensive disease small-cell lung cancer: JCOG 9702. Br J Cancer.

[31] Nagy-Mignotte H, Guillem P, Vignoud L (2012). Outcomes in recurrent small-cell lung cancer after one to four chemotherapy lines: a retrospective study of 300 patients. Lung Cancer.

[32] von Pawel J, Jotte R, Spigel DR (2014). Randomized phase III trial of amrubicin versus topotecan as second-line treatment for patients with small-cell lung cancer. J Clin Oncol.

[33] Kim YH, Mio T, Masago K, Irisa K, Sakamori Y, Mishima M (2010). Retrospective analysis of Japanese patients with relapse or refractory small-cell lung cancer treated with amrubicin hydrochloride. Oncol Lett.

[34] Okamoto I, Hamada A, Matsunaga Y (2006). Phase I and pharmacokinetic study of amrubicin, a synthetic 9-aminoanthracycline, in patients with refractory or relapsed lung cancer. Cancer Chemother Pharmacol.

