# Supplementary material for: A retrospective study of amrubicin monotherapy for the treatment of relapsed small cell lung cancer in elderly patients
Source: Cancer Chemother Pharmacol. 2017 Jul 31;80(3):615–22. doi: 10.1007/s00280-017-3403-9 (PMC5573757; doi:10.1007/s00280-017-3403-9)
Supplement: Supplementary file 1 — Supplementary material 1 (DOCX 24 kb) [file 280_2017_3403_MOESM1_ESM.docx]

Supplement Table 1. Univariate and multivariate analyses for progression-free survival and overall survival of good performance status (PS 0 and 1)

|  |  | Univariate analysis | | | Multivariate analysis | | |  | Univariate analysis | | | Multivariate analysis | | | |
| --- | --- | --- | --- | --- | --- | --- | --- | --- | --- | --- | --- | --- | --- | --- | --- |
|  |  | PFS | | | PFS | | |  | OS | | | OS | | | |
| **Factors** | **Median PFS (months)** | **HR** | **95% CI** | ***p*-value** | **HR** | **95% CI** | ***p*-value** | **Median OS (months)** | **HR** | **95% CI** | ***p*-value** | **HR** | **95% CI** | ***p*-value** |  |
| Sex |  |  |  |  |  |  |  |  |  |  |  |  |  |  |  |
| Male/female | 3.4/4.6 | 1.23 | 0.59-2.97 | 0.59 | 1.33 | 0.62-3.29 | 0.47 | 6.7/12.8 | 1.78 | 0.78-5.12 | 0.17 | 2.11 | 0.89-6.18 | 0.09 |  |
| Age (years) |  |  |  |  |  |  |  |  |  |  |  |  |  |  |  |
| 70-74/>=75 | 3.6/3.4 | 1.02 | 0.63-1.66 | 0.9 | 1.02 | 0.61-1.69 | 0.91 | 7.4/7.0 | 0.74 | 0.44-1.22 | 0.25 | 0.69 | 0.40-1.18 | 0.18 |  |
| Dose  (mg/m^2^ per day) |  |  |  |  |  |  |  |  |  |  |  |  |  |  |  |
| 30-35/40-45 | 3.0/4.3 | 1.22 | 0.74-2.03 | 0.42 | 1.32 | 0.78-2.28 | 0.29 | 5.7/8.7 | 1.37 | 0.83-2.30 | 0.21 | 1.46 | 0.86-2.52 | 0.15 |  |
| Relapse pattern |  |  |  |  |  |  |  |  |  |  |  |  |  |  |  |
| Sensitive/refractory | 4.3/2.7 | 0.57 | 0.35-0.95 | **0.031** | 0.57 | 0.34-0.95 | **0.032** | 8.8/5.8 | 0.77 | 0.47-1.27 | 0.3 | 0.71 | 0.42-1.21 | 0.21 |  |

PS, performance status; PFS, progression-free survival; OS, overall survival; HR, hazard ratio; 95% CI, 95% confidence interval

Bold-type *p* values are statistically significant (*p* < 0.05)

Supplement Table 2. Univariate and multivariate analyses for progression-free survival and overall survival of sensitive case

|  |  | Univariate analysis | | | Multivariate analysis | | |  | Univariate analysis | | | Multivariate analysis | | |
| --- | --- | --- | --- | --- | --- | --- | --- | --- | --- | --- | --- | --- | --- | --- |
|  |  | PFS | | | PFS | | |  | OS | | | OS | | |
| **Factors** | **Median PFS (months)** | **HR** | **95% CI** | ***p*-value** | **HR** | **95% CI** | ***p*-value** | **Median OS (months)** | **HR** | **95% CI** | ***p*-value** | **HR** | **95% CI** | ***p*-value** |
| Sex |  |  |  |  |  |  |  |  |  |  |  |  |  |  |
| Male/female | 4.0/3.4 | 0.82 | 0.36-2.19 | 0.66 | 0.7 | 0.27-2.04 | 0.5 | 7.8/6.8 | 0.98 | 0.43-2.64 | 0.97 | 0.85 | 0.33-2.48 | 0.75 |
| Age (years) |  |  |  |  |  |  |  |  |  |  |  |  |  |  |
| 70-74/>=75 | 3.5/4.4 | 1.15 | 0.60-2.15 | 0.65 | 1.3 | 0.63-2.71 | 0.46 | 5.8/8.8 | 1.29 | 0.66-2.44 | 0.43 | 1.68 | 0.81-3.46 | 0.15 |
| PS |  |  |  |  |  |  |  |  |  |  |  |  |  |  |
| 0-1/2-4 | 4.3/2.3 | 0.86 | 0.40-2.14 | 0.73 | 0.75 | 0.32-1.97 | 0.54 | 8.8/4.5 | 0.37 | 0.16-0.94 | **0.038** | 0.31 | 0.12-0.83 | **0.022** |
| Dose  (mg/m^2^ per day) |  |  |  |  |  |  |  |  |  |  |  |  |  |  |
| 30-35/40-45 | 3.4/4.3 | 0.98 | 0.52-1.89 | 0.96 | 0.96 | 0.48-1.92 | 0.9 | 7.0/7.8 | 1.23 | 0.65-2.40 | 0.51 | 1.18 | 0.58-2.40 | 0.64 |

PFS, progression-free survival; OS, overall survival; HR, hazard ratio; 95% CI, 95% confidence interval; PS, performance status

Bold-type *p* values are statistically significant (*p* < 0.05)

Supplement Table 3. Univariate and multivariate analyses for progression-free survival and overall survival of refractory case

|  |  | Univariate analysis | | | Multivariate analysis | | |  | Univariate analysis | | | Multivariate analysis | | |
| --- | --- | --- | --- | --- | --- | --- | --- | --- | --- | --- | --- | --- | --- | --- |
|  |  | PFS | | | PFS | | |  | OS | | | OS | | |
| **Factors** | **Median PFS (months)** | **HR** | **95% CI** | ***p*-value** | **HR** | **95% CI** | ***p*-value** | **Median OS (months)** | **HR** | **95% CI** | ***p*-value** | **HR** | **95% CI** | ***p*-value** |
| Sex |  |  |  |  |  |  |  |  |  |  |  |  |  |  |
| Male/female | 2.7/2.6 | 0.92 | 0.36-3.10 | 0.88 | 1.01 | 0.34-3.72 | 0.98 | 5.5/9.4 | 2.19 | 0.65-13.6 | 0.22 | 1.61 | 0.40-10.81 | 0.52 |
| Age (years) |  |  |  |  |  |  |  |  |  |  |  |  |  |  |
| 70-74/>=75 | 3.4/2.2 | 1 | 0.54-1.87 | 0.98 | 1.05 | 0.52-2.14 | 0.87 | 7.4/3.9 | 0.5 | 0.25-0.95 | **0.037** | 0.55 | 0.26-1.13 | 0.1 |
| PS |  |  |  |  |  |  |  |  |  |  |  |  |  |  |
| 0-1/2-4 | 2.7/2.7 | 0.89 | 0.45-1.92 | 0.76 | 0.94 | 0.45-2.12 | 0.88 | 5.8/3.6 | 0.51 | 0.23-1.23 | 0.12 | 0.5 | 0.22-1.23 | 0.46 |
| Dose  (mg/m^2^ per day) |  |  |  |  |  |  |  |  |  |  |  |  |  |  |
| 30-35/40-45 | 2.3/3.1 | 1.29 | 0.68-2.58 | 0.44 | 1.3 | 0.64-2.76 | 0.46 | 4.7/7.4 | 1.56 | 0.80-3.26 | 0.19 | 1.32 | 0.62-2.93 | 0.46 |

PFS, progression-free survival; OS, overall survival; HR, hazard ratio; 95% CI, 95% confidence interval; PS, performance status

Bold-type *p* values are statistically significant (*p* < 0.05)
